# Supportive care needs, quality of life and social support among elderly colorectal cancer patients undergoing chemotherapy: a longitudinal study

**DOI:** 10.3389/fonc.2024.1437888

**Published:** 2024-08-21

**Authors:** Siqin Lian, Xijie Hou, Weichen Liu, Ming Li, Guolian Chen, Ying Ling

**Affiliations:** ^1^ The Department of Nursing, First Affiliated Hospital, Guangxi Medical University, Nanning, Guangxi, China; ^2^ The Department of Blood Purification, First Affiliated Hospital, Guangxi Medical University, Nanning, Guangxi, China; ^3^ The Department of Oncology, First Affiliated Hospital, Guangxi Medical University, Nanning, Guangxi, China

**Keywords:** colorectal cancer, elderly patients, supportive care needs, quality of life, social support

## Abstract

**Objective:**

The purpose of this study is to examine the changes in supportive care needs, quality of life and social support during different chemotherapy cycles among elderly colorectal cancer patients.

**Methods:**

This prospective longitudinal study recruited 160 elderly colorectal cancer patients using convenience sampling at a hospital in Guangxi between August 2023 and April 2024. To assess supportive care needs, quality of life, and social support, we used a short form of the Supportive Care Needs Survey (SCNS-SF34), a Functional Assessment of Cancer Therapy-colorectal (FACT-C), and a perceived social support scale (PSSS) prior to chemotherapy, as well as after the first, third, and sixth cycles. Repeated measures analysis of variance was used to validate the changes over time in supportive care needs, quality of life, and social support.

**Results:**

155 participants completed all questionnaire sessions across the six cycles. From pre-chemotherapy until after the sixth cycle of chemotherapy, the extent of physical and daily living requirements among all respondents fluctuated between 47.23% and 88.26%, psychological needs ranged from 60.84% to 97.67%, patient care and support needs ranged from 83.75% to 99.35%, healthcare system and information needs varied from 85.98% to 99.00%, while the level of sexual needs decreased from 1.51% to 0.65%. The mean SCNS-SF34 scores for these participants ranged between 103.81 ± 2.28 and 144.10 ± 1.08. Significant increases over time were seen for all domains of SCNS-SF34 (*F*=126.99, 347.41, 65.00, 72.34, 160.15, *p*<0.001), keeping a clear upward trend, except for sexual needs(*F*=0.712, *p*=0.546). The mean FACT-T scores dropped from 68.80 ± 1.00 to 51.24 ± 1.40, while the mean PSSS scores dropped from 55.77 ± 0.83 to 43.28 ± 1.05. The scores of FACT-T and PSSS showed statistically significant differences (*F*=231.21, 112.28, *p*<0.001), maintaining clear downward trends.

**Conclusion:**

During chemotherapy, elderly colorectal cancer patients continue to require high levels of supportive care, while their quality of life and social support gradually decline. This study offers healthcare practitioners a foundational understanding to identify and address the supportive care needs of elderly colorectal cancer patients across various chemotherapy phases, which facilitates the development of tailored strategies aimed at enhancing patients’ quality of life.

## 1 Introduction

Colorectal cancer (CRC) remains a significant health concern worldwide, particularly among the elderly population. Based on the GLOBOCAN 2020 estimates, there were approximately 1.9 million new cases of CRC and 935,000 deaths in 2020, accounting for 10% and 9.4% of the global incidence and mortality of malignant tumors, ranking third and second in the incidence and mortality of all malignant tumors worldwide (1). In China, in the elderly population aged 60 and above, there were approximately 287,000 new cases of CRC and 156,000 deaths from CRC in 2016, accounting for 11.6% of the total new cases and 8.8% of the total deaths from cancer among the elderly population aged 60 and above (2). Furthermore, physiological changes associated with aging, frailty, medications, and multiple chronic conditions make managing and prognosing elderly CRC patients particularly difficult.

CRC patients who are diagnosed in early stages tend to have metastases, and nearly 50% of them will develop metastases in the future (3). The optimal treatment for these metastatic CRC patients remains perioperative chemotherapy or adjuvant chemotherapy with or without targeted agents before and after radical surgery (3). These radical treatments are associated with a variety of physical and psychosocial side-effects. The predominant adverse effects associated with chemotherapy include nausea, diarrhea, xerostomia, fatigue, somnolence, and anxiety, peaking in intensity during the immediate post-treatment period (4). With the aging population and increasing incidence of CRC among the elderly, the quality of life for this demographic is frequently impacted by both the disease symptoms and the treatment-related side effects (5). Numerous studies proved that evaluating and tackling unmet supportive care needs presents a chance to enhance health outcomes for all individuals impacted by cancer (6–8). Thus, recognizing the supportive care needs of older adults with CRC is essential for shaping healthcare policies, devising tailored interventions, and enhancing their overall quality of life.

The concept of supportive care needs originated from the Canadian Cancer Care Center in 1994 and has since become a widely used measure for evaluating the well-being of cancer patients worldwide (9). Since its introduction, extensive research has been conducted to improve patients’ quality of life and lessen the burden on caregivers by examining various aspects of supportive care needs, such as informational, emotional, psychosocial, practical, spiritual, and physical needs, aiming to identify the factors that influence these needs and develop customized interventions to meet them (10–13). However, there is limited research documenting the supportive care needs, quality of life, and social support of elderly CRC patients and how they evolve throughout the entire chemotherapy process. To provide continuous and individualized care, the supportive care needs, quality of life, and social support of elderly CRC patients should be investigated longitudinally to elicit the trajectories associated with these needs during chemotherapy. Therefore, this study aims to explore the variation in supportive care needs among elderly CRC patients undergoing chemotherapy across multiple treatment cycles, as well as their quality of life and level of social support.

## 2 Methods

### 2.1 Study design

A single-center prospective longitudinal study was conducted to investigate at four time points: from admission to one day before chemotherapy (T0), the last day of the intermission period after the first chemotherapy session (T1), the last day of the intermission period after the third chemotherapy session (T2), and the last day of the intermission period after the sixth chemotherapy session (T3).

### 2.2 Participants and setting

From August 1, 2023, to April 23, 2024, potential participants were screened and recruited at the Department of Oncology, Colorectal Surgery, and Day chemotherapy Center, of a tertiary hospital in Nanning, Guangxi, China, using a convenience sampling method. Patients were included if they met the following criteria: 1) diagnosed with colorectal cancer according to the diagnostic criteria outlined in the 2023 edition of the “National Health Commission of China CRC Diagnosis and Treatment Guidelines”; 2) age≥60 years old; 3) undergoing first-time chemotherapy for CRC; 4) having basic reading comprehension and communication skills. Patients were excluded if they met any of the following criteria: 1) severe mental or psychological disorders; 2) presence of other malignant tumors; 3) a history of radiotherapy, targeted therapy, or immunotherapy.

Based on Barcikowski and Robey’s sample size estimation table for a single-group repeated measures design (14), this study involved four repeated measurements on the study participants, with an average correlation coefficient ρ=0.50 and a significance level α= 0.05. Considering a dropout rate of 10% while ensuring 1-β=0.80, the minimum required sample size at baseline is 157 cases.

### 2.3 Measurements

Demographic details included age, gender, educational background, marital status, occupation, primary caregiver, residency, living arrangement, per capita monthly household income, medical insurance, and daily activity functionality. Disease characteristics included diagnostic time, stoma, type of surgery, diagnosis, chemotherapy regimen, the presence or absence of metastasis and comorbidities, and tumor type.

We used the Supportive Care Needs Survey-Short Form 34 (SCNS-SF34), which is a shortened version simplified by Boyes et al. (15)based on the questionnaire developed by Bonevski et al. (16), to investigate patients’ supportive care needs. This questionnaire is widely used in research on supportive care needs among cancer patients. The SCNS-SF34 is a self-reported questionnaire consisting of 34 items across 5 domains, including 10 items on psychological needs, 11 items on health system and information needs, 5 items on physical and daily living needs, 5 items on patient care and support needs, and 3 items on sexual needs. Scoring is done using a 5-point Likert scale, with higher scores indicating greater needs in that domain. Respondents were required to self-assess their level of need over the past month according to the survey items. The Cronbach’s α coefficients for the SCNS-SF34 was 0.850 in this study.

Functional Assessment of Cancer Therapy-colorectal (FACT-C) developed by Ward et al. (17) in 1999 and was used to investigate patients’ quality of life. The FACT-C comprises a general module for measuring cancer patients and a specific module for CRC, encompassing 36 items across 5 domains including Physical Well-being (PWB), Social/Family Well-being (SWB), Functional Well-being (FWB), Emotional Well-being (EWB), and CRC Additional Concerns (CCS). Each item is rated on a scale of 0 to 4, with higher scores indicating better quality of life. Positive items are scored directly from 0 to 4, while negative items are reverse-scored. The total score ranges from 0 to 136. Patients were asked to recall their health issues over the past 7 days. In this study, the Cronbach’s α coefficients for FACT-C was 0.825.

We also used the Perceived social support scale (PSSS) formulated by Gerg Zimet et al. (18) in 1988 to assess social support. The PSSS consists of two domains and 12 items in total, including family support and non-family support. Each item is scored on a scale of 1–7, with a total score ranging from 12–84, with higher scores indicating greater levels of social support. The Cronbach’s α coefficients for PSSS in our study was 0.865.

### 2.4 Data collection

Before the formal commencement of the study, we did a pre-survey to refine the study design. Researchers underwent intensive training to familiarize themselves with the precise content of the scales, clarify the research goals, and become proficient in the survey methods. After introducing the purpose and methods of the study to patients who met the inclusion criteria and obtaining their informed consent, researchers established personal files for patients and made thorough survey records. At T0, demographic and illness-related information, along with data on supportive care needs, quality of life, and social support survey data were gathered from patients. Subsequently, at T1, T2, and T3, data on supportive care needs, quality of life, and social support were collected. During hospitalization, patients were surveyed face-to-face using paper questionnaires, which were completed independently by the patients. Researchers patiently answered any questions patients had and collected the questionnaires on the spot. Following discharge, researchers conducted telephone follow-up sessions during which they queried patients using the research instruments and documented their responses. Each telephone follow-up session was limited to 20 minutes or less.

### 2.5 Ethical considerations

The study protocol was registered at https://www.medicalresearch.org.cn (2024-CR-037) and approved by the Ethics Committee of the First Affiliated Hospital of Guangxi Medical University (2024-K119–01). This study complied with the Declaration of Helsinki. All participants provided written informed consent. The participants were assured that they had the option to withdraw from the study or refuse to answer questionnaire questions at any point without affecting their treatment or care.

### 2.6 Statistical analyses

SPSS 25.0 statistical software was used to conduct a descriptive analysis of the socio-demographic data of the participants through frequency distribution, percentage, mean and standard deviation, median and quartile, and to calculate the degree of unmet supportive care needs of patients. Repeated measures analysis of variance (ANOVA) and pairwise comparison were employed to examine variations in patients’ supportive care needs, quality of life, and social support across different survey periods, with subsequent plotting of the trajectories by Excel 2021. If the data satisfied Mauchly’s sphericity test (P>0.05), the results were based on the hypothesis of sphericity test. If the data did not satisfy the sphericity test (P<0.05), the Pillai trajectory result from the multivariate test was used. The significance level of the test was α=0.05.

## 3 Results

### 3.1 Characteristics of participants

In total, 160 eligible participants were invited, of whom 155 finished this longitudinal study, yielding a response rate of 96.9%. Of the 5 patients who withdrew, 2 patients discontinued chemotherapy due to financial difficulty after the first cycle, 2 patients withdrew because of myelosuppression after the third cycle, and 1 patient died during the sixth cycle. The supportive care needs of these 5 patients were only tracked until they were excluded; there was no follow-up afterward. The average age of the 155 participants was 64.5 years. Most were under 65 years old, male, married/partnered, had a high school education or lower, received care from spouses/children, lived in rural areas, cohabited with others, and could independently carry out daily activities. Most patients had adenocarcinoma with less than 1 year of diagnosis, laparoscopic tumor radical surgery, chemotherapy with the Xelox regimen, and no comorbidities or stomas. Detailed demographic and clinical characteristics of the patients are presented in **Table 1**.

**Table 1:** Patient characteristics (N=155).

items		n(%)
Gender	Men	102(65.8)
	Women	53(34.2)
Age (mean ± SD)		64.5±4.5
Education level	Primary school	39(25.2)
	Secondary school	54(34.8)
	High school/technical secondary school	42(27.1)
	College or above	20(12.9)
Marital status	Married	147(94.8)
	Widowed	8(5.2)
Occupation	Farmer	87(56.1)
	Retired employee	55(35.5)
	Worker	13(8.4)
Primary caregiver	Spouse	92(59.4)
	Children	59(38.1)
	Own	4(2.6)
Residency	City	47(30.3)
	County or township	21(13.5)
	Rural areas	87(56.1)
Living status	Live alone	7(4.5)
	Not live alone	148(95.5)
Per capita monthly household income (yuan)	<2500	2(1.3)
	2500~4000	108(69.7)
	>4000	45(29)
Comorbidities	None	109(70.3)
	Hypertension	22(14.2)
	Diabetes mellitus(DM)	10(6.5)
	Coronary heart disease(CHD)	2(1.3)
	Hypertension + DM	7(4.5)
	Hypertension + CHD	4(2.6)
	Hypertension + DM +CHD	1(0.6)
Medical insurance	Medical insurance for urban residents	10(6.5)
	Medical insurance for rural residents	88(56.8)
	(retired)Worker with medical insurance	57(36.8)
Diagnostic time (year)	<1	140(90.3)
	1-3	14(9.0)
	>3	1(0.6)
function of daily activities(scores)	100	140(90.3)
	60-99	15(9.7)
Stoma	No	127(81.9)
	Yes	28(18.1)
Type of surgery	None	37(23.9)
	Endoscopic surgery	112(72.3)
	Laparotomy	6(3.9)
Diagnosis	Colon cancer	88(56.8)
	Rectal cancer	67(43.2)
Chemotherapy regime	Oxaliplatin + capecitabine (Xelox)	95(61.3)
	Xelox + tislelizumab	31(20.0)
	Xelox + Sintilimab	4(2.6)
	Xelox + bevacizumab	10(6.5)
	FOLFIRI+ cetuximab	7(4.5)
	mFOLFOX6+ cetuximab	7(4.5)
	Capecitabine	1(0.6)
Chemotherapy type	adjuvant	121 (78.1)
	neoadjuvant	34 (21.9)
Metastasis	No	83(53.5)
	Yes	72(46.5)
Cancer type	Adenocarcinoma	151(97.4)
	Signet-ring cell carcinoma	4(2.6)

### 3.2 Supportive care needs

#### 3.2.1 Prevalence of supportive care needs

Most patients indicated experiencing at least one moderate or high supportive care need during each assessment period. **Table 2** provides comprehensive summaries of the rates of unmet needs for each individual domain across all time-points.

**Table 2:** The unmet rates of all single-domain needs at all time-points (N=155).

Domains	No needs (%)	Needs satisfied (%)	Unmet needs (%)			
			Low needs	Moderate needs	High needs	Total
T0						
Physical and daily needs	26.06	26.71	12.13	12.65	22.45	47.23
Psychological needs	25.29	13.87	34.45	12.65	13.74	60.84
Sexual needs	98.28	0.22	1.51	0	0	1.51
Patient care and support needs	0.90	15.35	36.65	8.39	38.71	83.75
Health system and information needs	1.64	12.38	37.89	9.79	38.30	85.98
T1						
Physical and daily needs	18.71	8.77	30.70	10.32	31.48	72.50
Psychological needs	13.16	10.58	26.26	29.10	20.90	76.26
Sexual needs	97.63	1.08	1.29	0	0	1.29
Patient care and support needs	0.65	3.23	17.29	36.13	42.71	96.13
Health system and information needs	0.47	5.57	21.29	29.33	43.34	93.96
T2						
Physical and daily needs	10.45	8.52	10.19	21.42	49.42	81.03
Psychological needs	2.26	7.29	25.35	37.16	27.94	90.45
Sexual needs	98.06	1.29	0.65	0	0	0.65
Patient care and support needs	0	1.29	7.61	17.03	74.06	98.70
Health system and information needs	0	1.88	9.91	15.78	72.43	98.12
T3						
Physical and daily needs	8.65	3.10	7.35	17.81	63.10	88.26
Psychological needs	1.62	0.71	14.32	39.48	43.87	97.67
Sexual needs	99.14	0.22	0.65	0	0	0.65
Patient care and support needs	0%	0.65	2.06	5.16	92.13	99.35
Health system and information needs	0.06	0.94	3.75	6.63	88.62	99.00

N=155; The Supportive Care Needs Survey-Short Form 34 consists 5 domains, including 5 items on physical and daily living needs, 10 items on psychological needs, 3 items on sexual needs, 5 items on patient care and support needs, and 11 items on health system and information needs.

#### 3.2.2 Sum scores and changes over time

**Tables 3**–**5** displays the average scores and total scores of SCNS-SF34, FACT-C, and PSSS, along with their respective 95% confidence intervals and p-values across various time-points starting from baseline. Significant increases over time were seen for physical and daily living needs (*F*=126.99, *p*<0.001), psychological needs (*F*=347.41, *p*<0.001), patient care and support needs (*F*=65.00, *p*<0.001), health system and informational needs (*F*=72.34, *p*<0.001), and the sum scores of supportive care needs (*F*=160.15, *p*<0.001). Changes over time for sexual needs were not statistically significant (*F*=0.712, *p*=0.546). Patients exhibited notable declines in their overall quality of life and social support scores over time, with statistical significance observed (*F*=231.21, 112.28, *p*<0.001).

**Table 3:** The differences in scores for each domain and the total score of SCNS-SF34.

Time-points	$\bar{\chi }\pm S$	95%*CI*		F	P-value
		Lower bound	Upper bound		
Physical and daily needs					
T0	13.94±6.71	12.87	15.00	126.99	<0.001
T1	16.35±6.21	15.37	17.34		
T2	19.54±4.62	18.81	20.28		
T3	21.18±3.30	20.66	21.70		
Psychological needs					
T0	27.57±7.05	26.45	28.69	347.41	<0.001
T1	33.40±8.16	32.11	34.70		
T2	38.12±6.36	37.11	39.13		
T3	42.33±5.03	41.53	43.13		
Sexual needs					
T0	3.10±0.70	2.99	3.21	0.712	0.546
T1	3.10±0.73	2.99	3.23		
T2	3.08±0.59	2.98	3.17		
T3	3.05±0.49	2.97	3.12		
Patient care and support needs					
T0	18.43±5.73	17.52	19.34	65.00	<0.001
T1	20.85±4.27	20.17	21.53		
T2	23.19±3.39	22.66	23.73		
T3	24.44±2.07	24.11	24.77		
Health system and information needs					
T0	40.78±11.26	38.99	42.57	72.34	<0.001
T1	45.05±8.67	43.67	46.42		
T2	50.46±6.85	49.38	51.55		
T3	53.11±5.02	52.31	53.91		
Sum scores					
T0	103.81±2.28	99.31	108.32	160.15	<0.001
T1	118.76±2.00	114.81	122.72		
T2	134.40±1.46	131.51	137.29		
T3	144.10±1.08	141.97	146.24		

**Figure 1:**
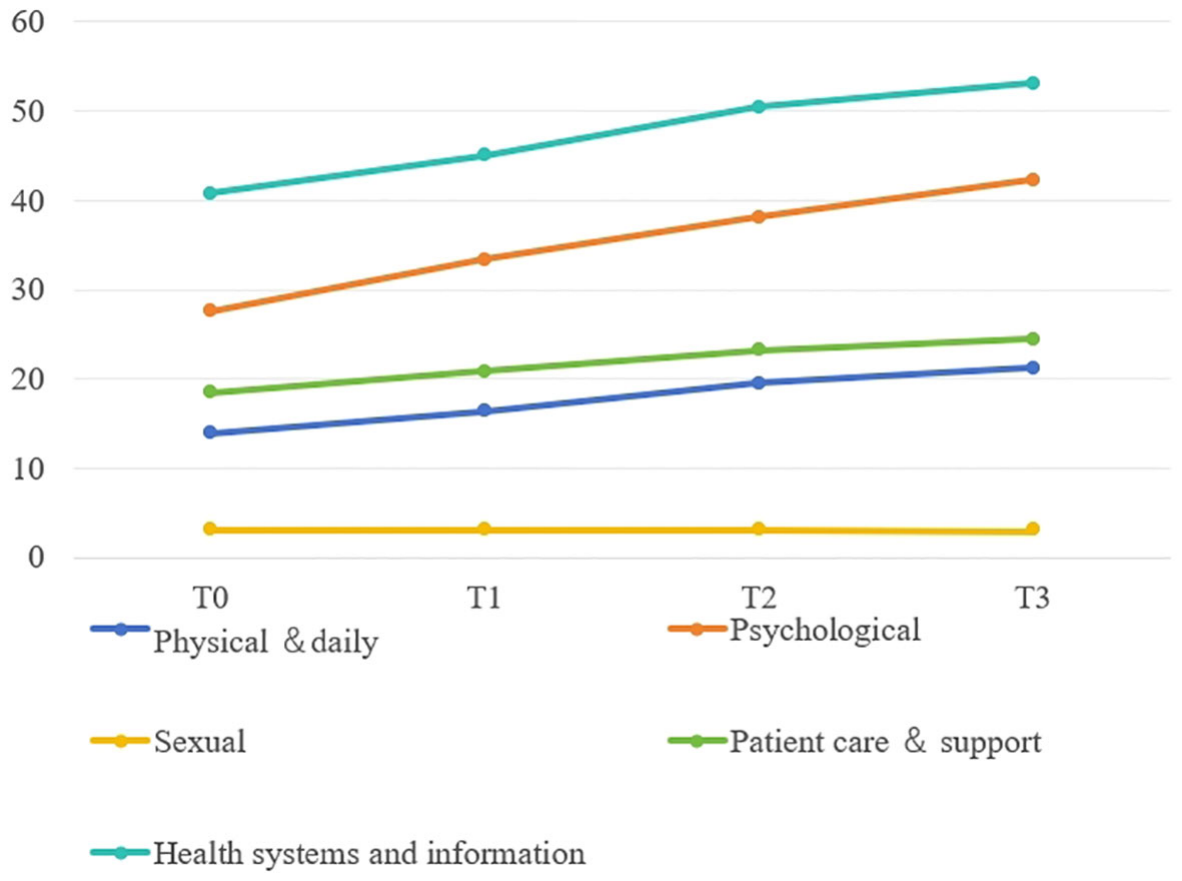
The estimated means and changes in scores for each domain of Supportive Care Needs Survey-Short Form 34. T0: from admission to one day prior to chemotherapy, T1-T3: the final day of the intermission period following the first, third, and sixth chemotherapy session.

**Figure 2:**
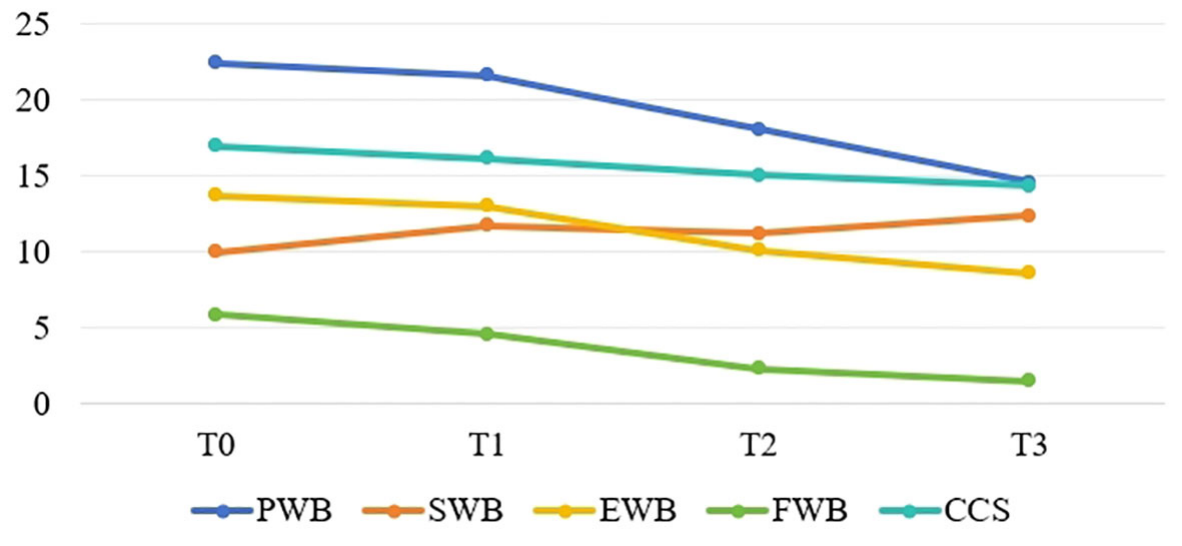
The estimated means and changes in scores for each domain of Functional Assessment of Cancer Therapy-colorectal. T0: from admission to one day prior to chemotherapy, T1-T3: the final day of the intermission period following the first, third, and sixth chemotherapy session. PWB, Physical Well-being; WSB, Social/Family Well-being; FWB, Functional Well-being; EWB, Emotional Well-being; CCS, Colorectal Additional Concerns.

**Figure 3:**
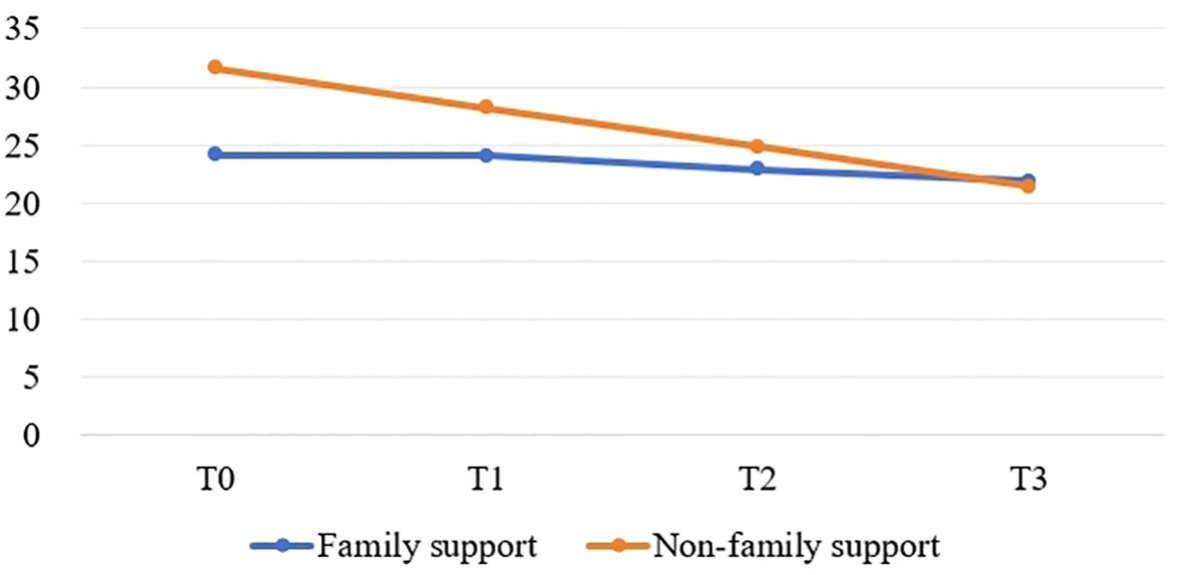
The estimated means and changes in scores for each domain of Perceived social support scale. T0: from admission to one day prior to chemotherapy, T1-T3: the final day of the intermission period following the first, third, and sixth chemotherapy session.

**Figure 4:**
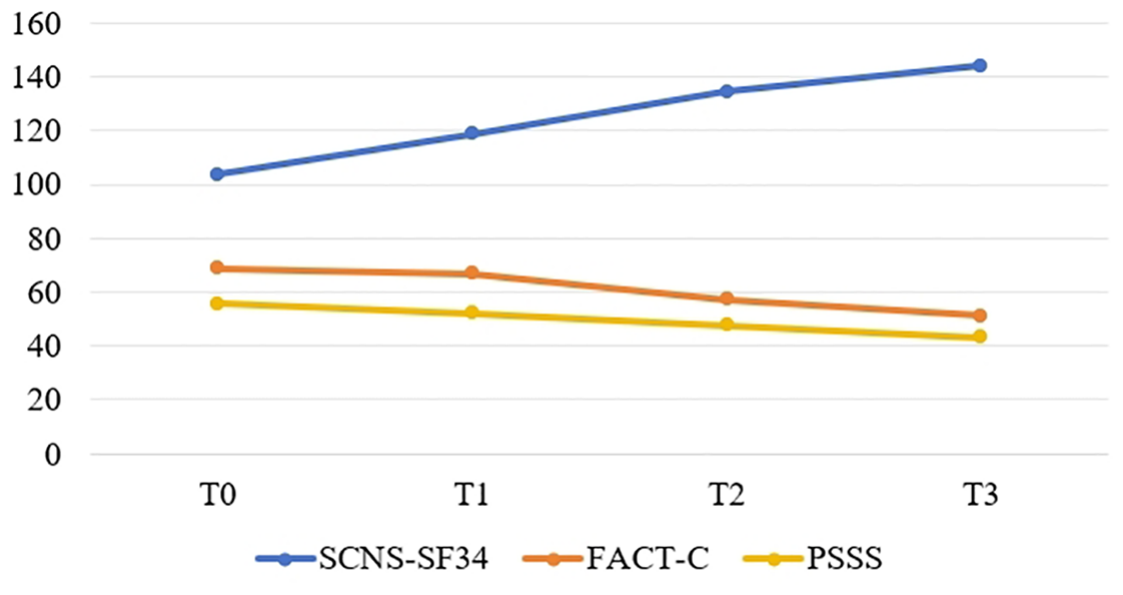
The estimated means and changes in the sum scores of SCNS-SF34, FACT-C and PSSS. T0: from admission to one day prior to chemotherapy, T1-T3: the final day of the intermission period following the first, third, and sixth chemotherapy session. SCNS-SF34, Supportive Care Needs Survey-Short Form 34; FACT-C, Functional Assessment of Cancer Therapy-colorectal; PSSS, Perceived social support scale.

**Figures 1**–**4** illustrates the estimated means, score fluctuations in each domain of SCNS-SF34, FACT-C, and PSSS, along with the sum scores. From T0 to T3, there was a noticeable rise in health system and information needs as well as psychological needs, whereas the increase in patient care and support needs, as well as physical and daily needs, was more gradual. Of FACT-C, the domain of PWB, EWB, FWB and CCS all showed a downward trend except SWB. As for PSSS, both family support and non-family support exhibited a decline over time.

**Table 4:** The differences in scores for each domain and the total score of FACT-C.

Time-points	$\bar{\chi }\pm S$	95%*CI*		F	P-value
		Lower bound	Upper bound		
PWB					
T0	22.42±4.20	21.75	23.09	905.16	<0.001
T1	21.56±4.89	20.79	22.34		
T2	18.02±4.67	17.28	18.76		
T3	14.57±6.72	13.50	15.63		
SWB					
T0	9.97±2.83	9.52	10.42	27.66	<0.001
T1	11.68±2.54	11.28	12.08		
T2	11.16±3.05	10.68	11.65		
T3	12.35±4.64	11.62	13.09		
EWB					
T0	13.66±4.00	13.02	14.29	170.88	<0.001
T1	12.99±4.90	12.22	13.77		
T2	10.90±4.41	10.20	11.60		
T3	8.55±4.05	7.91	9.20		
FEB					
T0	5.81±2.70	5.38	6.24	169.53	<0.001
T1	4.54±3.06	4.06	5.03		
T2	2.26±1.74	1.98	2.53		
T3	1.47±0.98	1.32	1.63		
CCS					
T0	16.94±3.83	16.33	17.55	33.96	<0.001
T1	16.10±4.18	15.43	16.76		
T2	15.00±2.12	14.66	15.34		
T3	14.29±3.23	13.78	14.80		
Sum scores					
T0	68.80±1.00	66.82	70.78	231.21	<0.001
T1	66.87±1.39	64.13	69.61		
T2	57.34±1.07	55.22	59.45		
T3	51.24±1.40	48.47	54.01		

PWB, Physical Well-being; WSB, Social/Family Well-being; FWB, Functional Well-being; EWB, Emotional Well-being; CCS, Colorectal Additional Concerns.

**Table 5:** The differences in scores for each domain and the total score of PSSS.

Time-points	$\bar{\chi }\pm S$	95%*CI*		F	P-value
		Lower bound	Upper bound		
Family support					
T0	24.16±3.30	23.64	24.69	16.58	<0.001
T1	24.10±3.94	23.48	24.73		
T2	22.87±3.62	22.30	23.45		
T3	21.87±3.40	21.24	22.51		
Non-family support					
T0	31.61±8.40	30.28	32.95	141.63	<0.001
T1	28.19±8.32	26.87	29.51		
T2	24.83±8.58	23.47	26.19		
T3	21.40±9.96	19.82	22.98		
Sum scores					
T0	55.77±0.83	54.14	57.41	112.28	<0.001
T1	52.29±0.84	50.63	53.95		
T2	47.70±0.89	45.95	49.46		
T3	43.28±1.05	41.20	45.35		

#### 3.2.3 The pairwise comparisons of SCNS-SF34, FACT-C, and PSSS at each time point

From pre-chemotherapy to after the sixth cycle of chemotherapy, the scores for supportive care needs gradually increased, while the scores for quality of life and social support gradually decreased. In pairwise comparisons at each time point, the differences were statistically significant (*p*<0.05). **Tables 6**–**8** display pairwise comparisons of the total scores for the SCNS-SF34, FACT-C, and PSSS at four time points from T0 to T3.

**Table 6:** Pairwise comparisons of SCNS-SF34 at various time points.

Time-point1	Time-point2	Mean difference	Standard error	95% CI of the difference	*p*-value
T0	T1	-14.948^*^	0.882	-16.69~ -13.21	<0.001
	T2	-30.587^*^	1.635	-33.82~ -27.36	<0.001
	T3	-40.290^*^	1.921	-44.09~ -36.50	<0.001
T1	T2	-15.639^*^	1.137	-17.89~ -13.39	<0.001
	T3	-25.342^*^	1.539	-28.38~ -22.30	<0.001
T2	T3	-9.703^*^	0.803	-11.29~ -8.12	<0.001

*The significance level of the mean difference is 0.05.

**Table 7:** Pairwise comparisons of FACT-C at various time points.

Time-point1	Time-point2	Mean difference	Standard error	95% CI of the difference	*p*-value
T0	T1	1.929^*^	0.825	0.30~ 3.56	<0.021
	T2	11.465^*^	0.752	9.98~ 12.95	<0.001
	T3	17.561^*^	1.141	15.31~ 19.82	<0.001
T1	T2	9.535^*^	0.459	8.63~ 10.44	<0.001
	T3	15.632^*^	0.612	14.42~ 16.84	<0.001
T2	T3	6.097^*^	0.501	5.11~ 7.09	<0.001

*The significance level of the mean difference is 0.05.

**Table 8:** Pairwise comparisons of PSSS at various time points.

Time-point1	Time-point2	Mean difference	Standard error	95% CI of the difference	*p*-value
T0	T1	3.484^*^	0.588	2.32~ 4.65	<0.001
	T2	8.071^*^	0.574	6.94~ 9.21	<0.001
	T3	12.497^*^	0.749	11.02~ 13.98	<0.001
T1	T2	4.587^*^	0.433	3.73~ 5.44	<0.001
	T3	9.013^*^	0.543	7.94~ 10.09	<0.001
T2	T3	4.426^*^	0.349	3.74~ 5.12	<0.001

*The significance level of the mean difference is 0.05.

## 4 Discussion

We investigated the supportive care needs, quality of life, and social support among elderly CRC patients from pre-chemotherapy to after the sixth cycle of chemotherapy. The results of this study showed that patient’s needs were constantly increasing throughout chemotherapy except sexual needs, with unmet needs accounted for a high percentage and did not improve over time, which was in line with previous findings. Armes et al. (19) revealed that there was evidence of ongoing unmet needs spanning 6 months, with 60% of patients who had more than five unmet needs at the end of their treatment showing no improvement. Lam et al. (20) similarly reported persistent unmet needs among CRC patients, beginning at diagnosis and continuing up to 12 months post-surgery. In our study, the most prevalent needs for information and psychological support were identified at the beginning of the study, and these needs remained consistently high even after six cycles of chemotherapy. As the demands for supportive care grew over time, there was a gradual decline in patients’ quality of life and social support.

Compared to other published data in supportive care needs (21–23), our study in elderly CRC patients showed higher sum scores for all supportive need domains. In the study, all patients underwent at least six cycles of chemotherapy. Chemotherapy brought about physical discomfort and side effects such as nausea, vomiting, and fatigue (24), which increased supportive care needs among patients. Additionally, the journey of cancer diagnosis and treatment triggered emotional fluctuations, highlighting the essential need for psychological support. Hui-Chun Hsu et al. (24) found that as the number of chemotherapy cycles increased, so did the occurrence of side effects such as peripheral numbness and hair loss. Danilo Galizia et al. (25) also confirmed that chemotherapy-induced side effects, such as appetite loss and taste disturbances, intensified with a rising number of chemotherapy cycles, resulting in heightened patient discomfort and requirements. Furthermore, chemotherapy disrupted patients’ daily lives, including work, family, and social activities. Nihal E. Mohamed et al. (26) found that patients who underwent chemotherapy after stoma urgently required improvements in their self-management skills for daily life and psychosocial adaptation. Therefore, medical staff should give more attention and support to meet care needs of elderly patients undergoing chemotherapy.

The enduringly high level of supportive care needs among elderly cancer patients may also stem from cognitive function decline during treatment. A previous investigation showed that cognitive function decline, including issues like memory loss or diminished concentration, might pose challenges for elderly patients (27), thereby amplifying their supportive care needs. Moreover, they were more likely to have comorbidities such as cardiovascular diseases, respiratory system diseases, and impaired liver or kidney function, increasing the treatment risks and result in poorer prognosis assessments (28), which leads to an increasing supportive care needs throughout the chemotherapy period.

In this study, patients’ information needs were the highest, which was consistent with previous studies (29, 30). Of the 155 participants, most participants(56.1%) in our study were farmers, and 87 patients(56.1%) lived in rural areas where information was relatively backward and thus requiring more health system and information support. Limited post-discharge contact with hospitals and constrained opportunities to acquire information may contribute to patients’ insufficient survival knowledge, resulting in unmet needs (31). Connecting with patients living in remote rural areas and maintaining the transmission of cancer-related health knowledge are key measures for medical staff to address patients’ health information needs. This study’s findings also indicated that patients, likely due to their older age, exhibited the lowest level of sexual need. It might also because patients thought sex was private and didn’t want to talk about. Another potential explanation could be that, influenced by Chinese traditional culture, patients may hesitate to openly express their sexual needs to others. Nevertheless, healthcare professionals should also consider educating patients about sexual health and encouraging their partners to address their sexual needs, recognizing that sexual well-being is integral to maintaining patients’ overall quality of life.

This study revealed that patients’ psychological needs ranked just below their health system and informational needs in terms of priority. The diagnosis of cancer induced significant emotional distress not only for the patients themselves but also for their families. Specifically, cancer and its treatments gave rise to considerable physical and psychological burdens that diminished the overall quality of life experienced by cancer survivors, both throughout and after their treatments (32). Moreover, psychological distress resulted in adverse repercussions, including but not limited to depression, sadness, anxiety, fear, worry, anger, or panic among individuals diagnosed with cancer (33). Therefore, it is necessary to pay attention to patients’ mental health in nursing work.

Our findings also suggested that patients’ quality of life gradually declined as chemotherapy progressed. A previous study conducted by Sodergren SC et al. (34) had the same finding with our study. The persistence of needs, without improvement after treatment completion, particularly in areas related to physical or daily functioning, psychological well-being, health system and information needs, was correlated with lower overall quality of life. Poorer FACT-C scores on the symptom assessments indicated the increasing odds of unmet needs. In particular, patients with stoma were found to be more prone to have unfulfilled needs and poorer quality of life across all domains (35). Prior researches validated unmet supportive care needs exerted a greater influence on the quality of life of cancer patients than socio-demographic or clinical factors (34, 36). Our research corroborated these findings and indicated that moderate to severe unmet needs were linked to a decrease in quality of life. Supportive care needs in all domains may correlated with diminished health and well-being, emphasizing the necessity of interventions in these areas.

Our study also found that patients’ social support decreased during chemotherapy, especially non-family support. A Study has shown that patients’ supportive care needs are significantly negatively correlated with patients’ social support (21), that is, the lower the social support patients receive, the higher the unmet supportive care needs. This is an issue that needs to be concerned by healthcare professionals. During chemotherapy, patients’ caregivers not only had the responsibility of caring for the patients but might also shoulder the burden of managing the entire family’s financial expenses. As a result, by the late stage of chemotherapy, the caregivers did not have much energy to spend on patients, so the family support of the patients in this study showed a downward trend. Non-family support includes support from friends and others. Treatment-induced weakness and low self-esteem in patients with stomas may affect patients’ social interaction, and reduced social interaction may be the reason for the decrease of non-family support. Studies have demonstrated that social support significantly contributes to alleviating physical, psychosocial and emotional challenges encountered by cancer patients throughout their illness trajectory. It expedites the healing journey, improves compliance with medical interventions, enhances quality of life, and extends life spans (37, 38). Therefore, medical staff should promote patient engagement in open communication with family and friends, facilitating the expression of internal concerns. Furthermore, they should provide guidance to patients’ social circles to offer increased care and support, ultimately mitigating patients’ physical and psychological distress.

## 5 Limitations

A limitation of our study was its single-center design. Moving forward, we planned to conduct multi-center research and increase the sample size to enhance the representativeness of our findings. Secondly, further exploration was needed regarding the relationship between supportive care needs, quality of life, and social support among elderly colorectal cancer patients in this study, as well as the factors influencing supportive care needs. Additionally, the follow-up period in this study only extended to after the sixth cycle of chemotherapy, failing to track the subsequent changes in the supportive care needs, quality of life, and social support of patients during their later stages of survival. The supportive care needs of patients who did not tolerate all chemotherapy cycles were only tracked until they were excluded and there was no follow-up afterward. They may have had higher supportive care needs. In future studies, we will improve by focusing more on these cancer patients.

## 6 Conclusions

We evaluated the changes in supportive care needs among 155 elderly CRC patients at different chemotherapy stages, alongside their quality of life and social support through questionnaires. We found that patients had high supportive care needs during treatment, which increased gradually over time. Meanwhile, their quality of life declined gradually, and their level of social support was relatively low. These research findings provide medical staff with a basis for identifying patients’ supportive care needs, developing corresponding care plans, and improving patients’ social support to meet their needs, thereby improving patients’ quality of life.

## Data Availability

The raw data supporting the conclusions of this article will be made available by the authors, without undue reservation.

## References

[1] Sung H Ferlay J Siegel RL Laversanne M Soerjomataram I Jemal A. Global cancer statistics 2020: GLOBOCAN estimates of incidence and mortality worldwide for 36 cancers in 185 countries. CA: Cancer J Clin. (2021) 71:209–49. 10.3322/caac.21660 33538338

[2] Zheng RS Zhang SW Sun KX Chen R Wang SM Li L. [Cancer statistics in China, 2016]. Zhonghua zhong liu za zhi [Chinese J oncology]. (2023) 45:212–20. 10.3760/cma.j.cn112152-20220922-00647 36944542

[3] Riesco-Martinez MC Modrego A Espinosa-Olarte P La Salvia A Garcia-Carbonero R. Perioperative chemotherapy for liver metastasis of colorectal cancer: lessons learned and future perspectives. Curr Treat options Oncol. (2022) 23:1320–37. 10.1007/s11864-022-01008-5 35980520

[4] Röhrl K Guren MG Småstuen MC Rustøen T. Symptoms during chemotherapy in colorectal cancer patients. Supportive Care cancer: Off J Multinational Assoc Supportive Care Cancer. (2019) 27:3007–17. 10.1007/s00520-018-4598-y 30607676

[5] Dekker E Tanis PJ Vleugels JLA Kasi PM Wallace MB. Colorectal cancer. Lancet (London England). (2019) 394:1467–80. 10.1016/S0140-6736(19)32319-0 31631858

[6] Chen SC Chiou SC Yu CJ Lee YH Liao WY Hsieh PY. The unmet supportive care needs-what advanced lung cancer patients’ caregivers need and related factors. Supportive Care cancer: Off J Multinational Assoc Supportive Care Cancer. (2016) 24:2999–3009. 10.1007/s00520-016-3096-3 26872793

[7] Post L Liefbroer AI. Reducing distress in cancer patients-A preliminary evaluation of short-term coaching by expert volunteers. Psycho-oncology. (2019) 28:1762–6. 10.1002/pon.5111 PMC6771510 31107998

[8] Walshe C Roberts D. Peer support for people with advanced cancer: a systematically constructed scoping review of quantitative and qualitative evidence. Curr Opin supportive palliative Care. (2018) 12:308–22. 10.1097/SPC.0000000000000370 29979318

[9] Fitch MI. Supportive care framework. Can Oncol Nurs J = Rev Can Nurs oncologique. (2008) 18:6–24. 10.5737/1181912x181614 18512565

[10] Shinall MC Jr. Ely EW Diehl C Beskow LM. Patient perspectives on perioperative supportive care needs surrounding major abdominal operations for cancer. Ann Surg Oncol. (2023) 30:2597–605. 10.1245/s10434-022-12895-1 PMC10184497 36463355

[11] Miniotti M Bassino S Fanchini L Ritorto G Leombruni P. Supportive care needs and the impact of loss of functioning and symptom burden on the quality of life in patients with advanced colorectal cancer. Oncol Res Treat. (2022) 45:262–71. 10.1159/000521753 34983050

[12] Kim H Yoo YS. Factors influencing supportive care needs of colorectal cancer survivors. Asian Nurs Res. (2021) 15:60–6. 10.1016/j.anr.2020.11.003 33249141

[13] Sussman J Bainbridge D Whelan TJ Brazil K Parpia S Wiernikowski J. Evaluation of a specialized oncology nursing supportive care intervention in newly diagnosed breast and colorectal cancer patients following surgery: a cluster randomized trial. Supportive Care cancer: Off J Multinational Assoc Supportive Care Cancer. (2018) 26:1533–41. 10.1007/s00520-017-3981-4 29189967

[14] Barcikowski RS Randall RR. Sample size selection in single group repeated measures analysis. J Educational Stat. (1985) 10:243–61.

[15] Boyes A Girgis A Lecathelinais C. Brief assessment of adult cancer patients’ perceived needs: development and validation of the 34-item Supportive Care Needs Survey (SCNS-SF34). J Eval Clin practice. (2009) 15:602–6. 10.1111/j.1365-2753.2008.01057.x 19522727

[16] Bonevski B Sanson-Fisher R Girgis A Burton L Cook P Boyes A. Evaluation of an instrument to assess the needs of patients with cancer. Supportive Care Review Group. Cancer. (2000) 88:217–25. 10.1002/(sici)1097-0142(20000101)88:1<217::aid-cncr29>3.0.co;2-y 10618626

[17] Ward WL Hahn EA Mo F Hernandez L Tulsky DS Cella D. Reliability and validity of the Functional Assessment of Cancer Therapy-Colorectal (FACT-C) quality of life instrument. Qual Life research: an Int J Qual Life aspects treatment Care rehabilitation. (1999) 8:181–95. 10.1023/a:1008821826499 10472150

[18] Zimet GD Powell SS Farley GK Werkman S Berkoff KA. Psychometric characteristics of the multidimensional scale of perceived social support. J Pers Assess. (1990) 55:610–7. 10.1080/00223891.1990.9674095 2280326

[19] Armes J Crowe M Colbourne L Morgan H Murrells T Oakley C. Patients’ supportive care needs beyond the end of cancer treatment: a prospective, longitudinal survey. J Clin oncology: Off J Am Soc Clin Oncol. (2009) 27:6172–9. 10.1200/JCO.2009.22.5151 19884548

[20] Lam WW Law WL Poon JT Fong D Girgis A Fielding R. A longitudinal study of supportive care needs among Chinese patients awaiting colorectal cancer surgery. Psycho-oncology. (2016) 25:496–505. 26333916 10.1002/pon.3946

[21] Xiangting Y Meichun Z Huiying Q. Supportive care needs and related factors among colorectal cancer patients with stoma in the postoperative rehabilitation period from a bio-psycho-social perspective: a cross-sectional study. Supportive Care cancer: Off J Multinational Assoc Supportive Care Cancer. (2023) 31:599. 10.1007/s00520-023-08067-w 37770807

[22] Lam WW Tsang J Yeo W Suen J Ho WM Yau TK. The evolution of supportive care needs trajectories in women with advanced breast cancer during the 12 months following diagnosis. Supportive Care cancer: Off J Multinational Assoc Supportive Care Cancer. (2014) 22:635–44. 10.1007/s00520-013-2018-x 24158684

[23] Giuliani ME Milne RA Puts M Sampson LR Kwan JY Le LW. The prevalence and nature of supportive care needs in lung cancer patients. Curr Oncol (Toronto Ont). (2016) 23:258–65. 10.3747/co.23.3012 PMC4974033 27536176

[24] Hsu HC Tsai SY Wu SL Jeang SR Ho MY Liou WS. Longitudinal perceptions of the side effects of chemotherapy in patients with gynecological cancer. Supportive Care cancer: Off J Multinational Assoc Supportive Care Cancer. (2017) 25:3457–64. 10.1007/s00520-017-3768-7 28634657

[25] Galizia D Milani A Geuna E Martinello R Cagnazzo C Foresto M. Self-evaluation of duration of adjuvant chemotherapy side effects in breast cancer patients: A prospective study. Cancer Med. (2018) 7:4339–44. 10.1002/cam4.1687 PMC6144000 30030895

[26] Mohamed NE Shah QN Kata HE Sfakianos J Given B. Dealing with the unthinkable: bladder and colorectal cancer patients’ and informal caregivers’ Unmet needs and challenges in life after ostomies. Semin Oncol nursing. (2021) 37:151111. 10.1016/j.soncn.2020.151111 33423864

[27] Lange M Giffard B Noal S Rigal O Kurtz JE Heutte N. Baseline cognitive functions among elderly patients with localised breast cancer. Eur J Cancer (Oxford England: 1990). (2014) 50:2181–9. 10.1016/j.ejca.2014.05.026 24958735

[28] Siegel RL Miller KD Fedewa SA Ahnen DJ Meester RGS Barzi A. Colorectal cancer statistics, 2017. CA: Cancer J Clin. (2017) 67:177–93. 10.3322/caac.21395 28248415

[29] Al-Husban RY Obeidat R Shamieh O. Unmet supportive care needs of Jordanian patients with colorectal cancer: A cross-sectional survey. Asia-Pacific J Oncol nursing. (2021) 8:565–72. 10.4103/apjon.apjon-2110 PMC8420928 34527787

[30] Pembroke M Bradley J Nemeth LS. Breast cancer survivors’ Unmet needs after completion of radiation therapy treatment. Oncol Nurs forum. (2020) 47:436–45. 10.1188/20.ONF.436-445 32555557

[31] Chae BJ Lee J Lee SK Shin HJ Jung SY Lee JW. Unmet needs and related factors of Korean breast cancer survivors: a multicenter, cross-sectional study. BMC cancer. (2019) 19:839. 31455311 10.1186/s12885-019-6064-8 PMC6712787

[32] Courneya KS Friedenreich CM Sela RA Quinney HA Rhodes RE Handman M. The group psychotherapy and home-based physical exercise (group-hope) trial in cancer survivors: physical fitness and quality of life outcomes. Psycho-oncology. (2003) 12:357–74. 10.1002/pon.658 12748973

[33] Kim JY Lee MK Lee DH Kang DW Min JH Lee JW. Effects of a 12-week home-based exercise program on quality of life, psychological health, and the level of physical activity in colorectal cancer survivors: a randomized controlled trial. Supportive Care cancer: Off J Multinational Assoc Supportive Care Cancer. (2019) 27:2933–40. 10.1007/s00520-018-4588-0 30564936

[34] Sodergren SC Wheelwright SJ Permyakova NV Patel M Calman L Smith PWF. Supportive care needs of patients following treatment for colorectal cancer: risk factors for unmet needs and the association between unmet needs and health-related quality of life-results from the ColoREctal Wellbeing (CREW) study. J Cancer survivorship: Res practice. (2019) 13:899–909. 10.1007/s11764-019-00805-6 PMC6881415 31512164

[35] Taylor C. Body image concerns after colorectal cancer surgery. Br J Nurs (Mark Allen Publishing). (2015) 24:S8, s10–2, s4. 10.12968/bjon.2015.24.Sup10.S8 26018183

[36] Okediji PT Salako O Fatiregun OO. Pattern and predictors of unmet supportive care needs in cancer patients. Cureus. (2017) 9:e1234. 28620565 10.7759/cureus.1234 PMC5467772

[37] Aydın Sayılan A Demir Doğan M. Illness perception, perceived social support and quality of life in patients with diagnosis of cancer. Eur J Cancer Care. (2020) 29:e13252. 10.1111/ecc.13252 32495471

[38] Yilmaz MS Piyal B Akdur R. Social support and quality of life in a group of cancer patients (Ankara, Turkey). Turkish J Med Sci. (2017) 47:732–7. 10.3906/sag-1508-42 28618743

